# PAVSAT: an automated blood vessel analysis tool using deep learning-based segmentation and image processing

**DOI:** 10.1186/s12859-026-06505-0

**Published:** 2026-06-22

**Authors:** Ryozo Ishida, Naoi Hosoe, Anna Shimizu, Kazuhiro Takara, Yumiko Hayashi, Lamri Lynda, Hiroyasu Kidoya

**Affiliations:** 1 Department of Integrative Vascular Biology, Faculty of Medical Sciences, University of Fukui, Fukui, 910-1193 Japan; 2 Tenure-Track Program for Innovative Research, University of Fukui, Fukui, 910-1193 Japan; 3 Otorhinolaryngology-Head and Neck Surgery, Faculty of Medicine, University of Fukui, Fukui, 910-1193 Japan

**Keywords:** Blood vessel analysis, Deep learning, Image processing, Vascular morphology, Graph-based analysis

## Abstract

**Background:**

Accurate blood vessel morphology analysis is essential for understanding vascular system development and pathology. Although advanced imaging technologies. have enabled the rapid acquisition of complex vascular images, quantitative analysis methods have not kept pace. Importantly, current methods cannot accurately represent vessels with complex curved structures and variable diameters, and the existing software lacks efficient automation for large datasets.

**Results:**

In this study, we developed PAVSAT (Python-based Auto Vessel Segmentation Analysis Tool), an automated computational system for quantifying vascular structures by integrating deep learning-based segmentation, image processing, and graph theory. The system employs a YOLOv8 architecture trained on confocal immunofluorescence microscopy images of CD31-stained vascular structures, followed by skeletonization to extract the vessel centerlines. A novel branch-point detection algorithm identifies bifurcations by analyzing local connectivity patterns and performs dense, segment-wise vessel diameter profiling through repeated perpendicular sampling along the locally estimated vessel orientation. To mitigate systematic detection errors at tile boundaries, we implemented a boundary-aware overlapping tiling scheme in which images are processed with spatial offsets and outputs merged to ensure that each sinusoid is analyzed under optimal central-tile conditions. Ablation analysis confirmed that this overlapping integration substantially reduced undetected vessels (by 40.5 and 81.0% in two independent samples) with only marginal increases in false-positive detections (2.2 and 6.3%, respectively), indicating a clearly favorable trade-off between sensitivity and specificity. A graph-based representation converts the vascular structures into nodes and edges for network analysis. Validation against expert manual measurements demonstrated detection rates of 91–98% and measurement accuracy of 89–93% within 10 pixels of the manual measurements, with no detectable systematic bias between automated and manual diameters (mean difference < 0.3 pixels, paired t-test and Wilcoxon *p* > 0.2 for both samples) and strong agreement (Pearson r > 0.94).

**Conclusions:**

PAVSAT successfully identified complex branching patterns, measured the diameters along curved vessels, and generated quantitative data suitable for large-scale studies, thereby facilitating vascular biology, development, and pathology research.

**Supplementary Information:**

The online version contains supplementary material available athttps://doi.org/10.1186/s12859-026-06505-0.

## Background

Vascular networks provide essential infrastructure for oxygen and nutrient delivery throughout tissues and are critical to both physiological and pathological processes [1, 2]. Detailed analyses of blood vessel morphology, distribution, and connectivity are crucial for understanding vascular development, remodeling, and dysfunction under various conditions including ischemia, tumor angiogenesis, and vascular malformations [3, 4]. Recent clinical and translational studies have demonstrated that quantitative analysis of vascular microstructure derived from endothelial markers, such as CD31, contributes to objective disease evaluation models and therapy-response assessment, including the characterization of vessel normalization following anti-angiogenic treatment [5]. In this context, computational modeling approaches leveraging CD31-derived vascular features are increasingly used to link microstructural remodeling with treatment efficacy and disease progression [6]. Accordingly, developing more detailed quantitative descriptors and novel vascular metrics is expected to enhance the clinical utility of vascular analysis pipelines and improve model-based disease stratification. Blood vessel architecture presents analytical challenges because vessels do not follow linear paths but instead curve and branch in complex patterns that adapt to local tissue environments [7]. Traditional measurement methods that approximate vessels as straight or uniformly thick structures fail to capture this complexity, resulting in substantial information loss. Historically, vascular analysis relied on manual or semi-manual stereological measurements, which are time-consuming, prone to observer bias, and limited in capturing spatial heterogeneity and network-level organization [8, 9].

Existing computational approaches for vascular analysis can be categorized by imaging modality and data dimensionality. For two-dimensional fluorescence and confocal microscopy images, simple computational tools such as ImageJ with various plugins enable basic quantification of vessel length and branching patterns; however, these approaches assume uniform vessel diameters and fail to capture natural morphological variability [10–12]. Classical image processing techniques, including thresholding and region-growing algorithms, have also been applied to vessel segmentation in such images, but they often struggle in low-contrast regions and at complex vessel crossings. AngioTool represents an incremental advancement by incorporating vessel enhancement filters to improve detection [13], while REAVER integrates automated analysis with manual curation through an editable workflow [14]. Nevertheless, both tools remain dependent on threshold-based segmentation strategies, which may overlook fine or low-intensity vascular structures. These limitations have been highlighted in comparative reviews of vessel segmentation techniques applied to microscopy data [15]. In contrast, volumetric imaging modalities, such as angiography, micro-computed tomography, and magnetic resonance angiography (MRA), have driven the development of three-dimensional vessel segmentation methods based on machine learning. Deep learning architectures, particularly U-Net-derived models, have become a standard backbone for vessel segmentation in these contexts [16]. Approaches such as DeepVesselNet extend this paradigm by jointly predicting vessel segmentation, centerlines, and bifurcation points in three-dimensional angiographic volumes [17]. A growing body of work in MRA focuses on automated segmentation and volumetric quantification of major cerebral vessels and intracranial aneurysms using advanced convolutional neural networks, including attention-based, multi-scale, and dilated architectures [18–23]. These methods demonstrate high accuracy and robustness in clinical neuroimaging applications, enabling vessel-level or lesion-level assessment in pre- and post-treatment settings. While these approaches improve connectivity preservation and segmentation accuracy, their primary objective is volumetric segmentation and global morphometric assessment of relatively large-caliber vessels. Consequently, they are designed for three-dimensional datasets with high signal-to-noise ratios and do not directly address fine-grained diameter profiling along curved vessel paths in high-resolution two-dimensional fluorescence or confocal microscopy images. More recently, tissue-specific deep learning approaches, particularly for retinal imaging, have demonstrated strong performance in vessel segmentation and quantitative analysis [15, 24–28]. Despite these advances, many methods are optimized for specific imaging conditions or anatomical sites, limiting general applicability across diverse tissues and experimental settings. Consequently, a versatile framework capable of robustly analyzing complex vascular morphologies in high-resolution fluorescence microscopy images remains lacking. Importantly, recent technological advances have revolutionized vascular imaging capabilities. Two-photon excitation microscopy enables deep-tissue imaging with minimal photodamage [29–31], whereas light-sheet microscopy allows rapid acquisition of large tissue volumes with cellular resolution [32, 33]. Additionally, novel fluorescent probes with high intensity and stability, combined with tissue clearing techniques, have significantly improved the ability to visualize complete vascular networks within tissues [34, 35]. Despite these imaging advances, computational tools for vascular analysis have not kept pace [36].

Current software solutions employ simplified models that approximate vessels as uniform, straight geometric segments, thereby failing to account for natural vessel morphology variations. Furthermore, several existing tools require extensive manual intervention, creating bottlenecks in analysis workflows and introducing operator-dependent variability. The gap between imaging capabilities and analytical tools has created an urgent need for sophisticated computational approaches for handling the complexity and scale of modern vascular imaging data [37]. Notably, deep learning and advanced image processing techniques offer promising solutions to these challenges, potentially capturing the full range of vascular morphologies without relying on simplified models. Here, we present a novel computational approach for the automated analysis of blood vessel images that addresses the limitations of the current methods. The integrated pipeline leverages deep learning for initial segmentation, advanced image processing for feature extraction, and graph-based analysis for characterizing network properties, thereby providing researchers with quantitative vascular analysis tools.

## Implementation

### System overview

The PAVSAT (Python-based Auto Vessel Segmentation Analysis Tool) system integrates deep learning-based segmentation, image processing, and graph theory into an automated pipeline. The workflow consists of (1) YOLOv8-based vessel segmentation, (2) skeletonization and branch point detection, (3) vessel diameter measurement, and (4) graph-based network analysis.

### Preparing the image dataset

The vascular images used in this study were obtained through collaboration with the Department of Otorhinolaryngology-Head & Neck Surgery at the University of Fukui. Images were acquired from mouse nasal septum tissue using whole-mount immunofluorescence microscopy. Female BALB/c mice (*n* = 8) (Sankyo Labo Service Corporation, Shizuoka, Japan) were housed under a 12-h light/dark cycle with unrestricted access to food and water. Blood vessels were visualized using CD31 and endomucin immunofluorescence labeling. Images were captured using a Leica STELLARIS 5 confocal microscope. All animal experiments were conducted in accordance with protocols approved by the Institutional Animal Care and Use Committee of the University of Fukui (approval numbers: R04091, R05047, R06049, R07047). Mice were euthanized using carbon dioxide inhalation following institutional guidelines. All mice used in this study were healthy and had no induced pathology, genetic modification, or experimental intervention affecting the vasculature.

### Deep learning-based segmentation and image processing

**Fig. 1:**
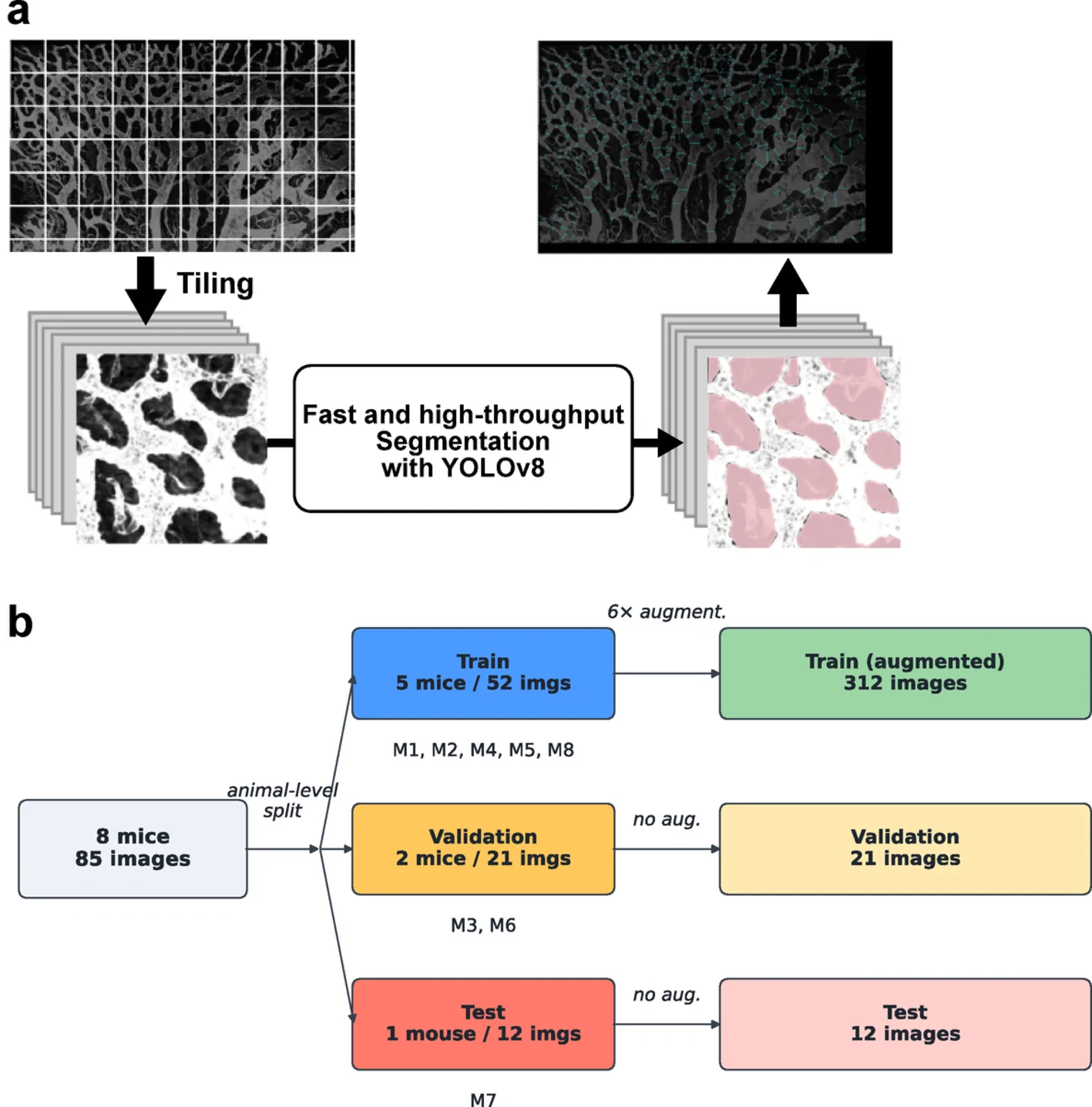
YOLOv8 segmentation framework and animal-level dataset partitioning. **a** Overview of the segmentation component within the proposed framework, illustrating the tile-based strategy in which numerous small image patches are processed as independent segmentation tasks, prioritizing efficient high-throughput analysis of sinusoid regions over highly complex anatomical modeling. **b** Schematic diagram of animal-level dataset partitioning, in which 85 images from 8 mice were split into training (5 mice, 52 images; M1, M2, M4, M5, and M8), validation (2 mice, 21 images; M3 and M6), and test (1 mouse, 12 images; M7) subsets, with no mouse shared across subsets. The training subset was then augmented 6 × by horizontal flipping, vertical flipping, and 90°/180°/270° rotation, yielding 312 images; the validation and test subsets were used without augmentation

In this study, YOLOv8 was selected as the segmentation backbone because it meets the practical requirements of our analysis pipeline. YOLOv8 provides a favorable balance between inference speed and segmentation accuracy, enabling efficient high-throughput processing while remaining deployable on standard laboratory hardware (Ultralytics documentation). The model supports integration into Python-based workflows, fine-tuning from pretrained weights, and imposes modest computational demands, ensuring accessibility for users without specialized infrastructure. These characteristics are well suited to our tile-based strategy, described later in this section, in which a large number of image patches must be processed efficiently (Fig. 1a). The present task focuses on sinusoid regions with relatively simple geometric structures compared with more complex anatomical targets; therefore, highly sophisticated segmentation capability is unnecessary, and moderate accuracy combined with rapid throughput is sufficient for the downstream quantitative analysis performed in this study.

**Fig. 2:**
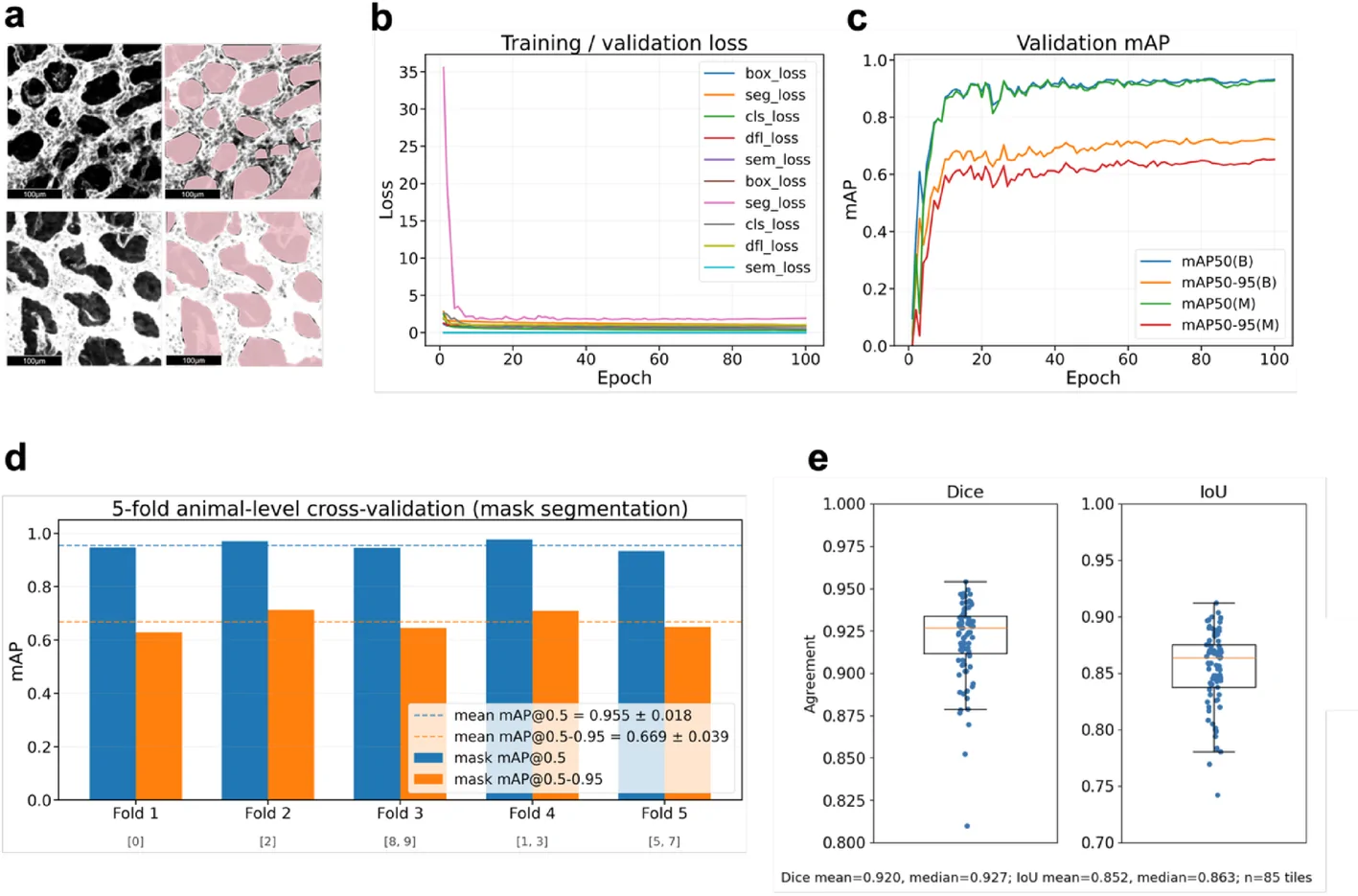
Annotation examples and model performance. **a** Examples of sinusoid annotation, together with the augmentation scheme applied only to the training subset (horizontal flipping, vertical flipping, and 90°/180°/270° rotation; 6 × expansion, 52 → 312 images). **b** Training and validation loss curves over 100 epochs (best epoch: 76), exhibiting model convergence. **c** Training and validation mAP curves over the same 100 epochs, reaching a final mask mAP@0.5 of 0.916 and mAP@0.5–0.95 of 0.646 on the held-out test set. **d** Five-fold cross-validation performance at the animal level using GroupKFold, showing mean mask mAP@0.5 and mAP@0.5–0.95 across folds with minimal inter-fold variance, indicating stable and animal-partition-independent segmentation performance. **e** Intra-observer variability analysis based on repeated annotation of 85 images after a 6-month interval, demonstrating high agreement quantified by mean Dice and IoU metrics and supporting annotation reliability. mAP, mean average precision

The original images were partitioned at the animal level into training (*n* = 52 images from 5 mice: M1, M2, M4, M5, and M8), validation (*n* = 21 images from 2 mice: M3 and M6), and test (*n* = 12 images from 1 mouse: M7) subsets (Fig. 1b). No mouse appeared in more than one subset, thereby avoiding inter-individual data leakage that would arise from image-level partitioning. We annotated specialized vascular channels, called sinusoids, in 85 images of the mouse nasal septum from the image dataset, and augmented only the training subset after the animal-level split, expanding it from 52 to 312 images (factor of 6) via horizontal flipping, vertical flipping, and 90°/180°/270° rotation (Fig. 2a).

Applying augmentation only after splitting ensured that augmented variants of the same base image were never distributed across different subsets, preventing information leakage. Training metrics demonstrated improvement over 100 epochs (best epoch: 76), with the loss curves stabilizing, indicating model convergence (Fig. 2b, c). On the held-out test set (mouse M7, unseen during training and validation), the final model achieved a mask mean average precision (mAP) of 0.916 at an IoU threshold of 0.5 and 0.646 at IoU 0.5–0.95, demonstrating robust vessel detection capability on previously unseen individuals. To further evaluate model robustness and generalizability beyond a single train–validation split, we conducted five-fold cross-validation at the animal level using GroupKFold on the 85 base images, such that the validation mice in each fold were strictly separated from the corresponding training mice to avoid inter-individual data leakage. Across the five folds, the mean mask mAP@0.5 was 0.955 ± 0.018, and the mean mask mAP@0.5–0.95 was 0.669 ± 0.039. Performance remained consistent across folds, with minimal inter-fold variance, indicating stable segmentation behavior and suggesting that the observed detection performance was not dependent on a specific train–test animal partition (Fig. 2d). To further evaluate the reliability of the ground-truth annotations, we assessed intra-observer variability by re-annotating the same 85 original images after an interval exceeding 6 months using an independent annotation platform. Agreement between the initial and repeated annotations was quantified using pixel-wise Dice and IoU metrics derived from merged segmentation masks for each image. Across the 85 matched images, the mean Dice coefficient was 0.919 ± 0.023, and the mean IoU was 0.852 ± 0.038. No systematic bias in vessel coverage was observed between the two annotation rounds. These findings indicate high intra-observer consistency and support the reliability of the ground-truth annotations used for model training and evaluation (Fig. 2e). To improve interpretability of the deep learning component, we performed layer-wise feature visualization and gradient-weighted class activation mapping (Grad-CAM) analysis (Additional file 1). The results revealed hierarchical feature encoding from vessel edges to continuous tubular structures, with attention concentrated along vessel contours rather than background regions. This observation further supports the robustness of the segmentation component.

**Fig. 3:**
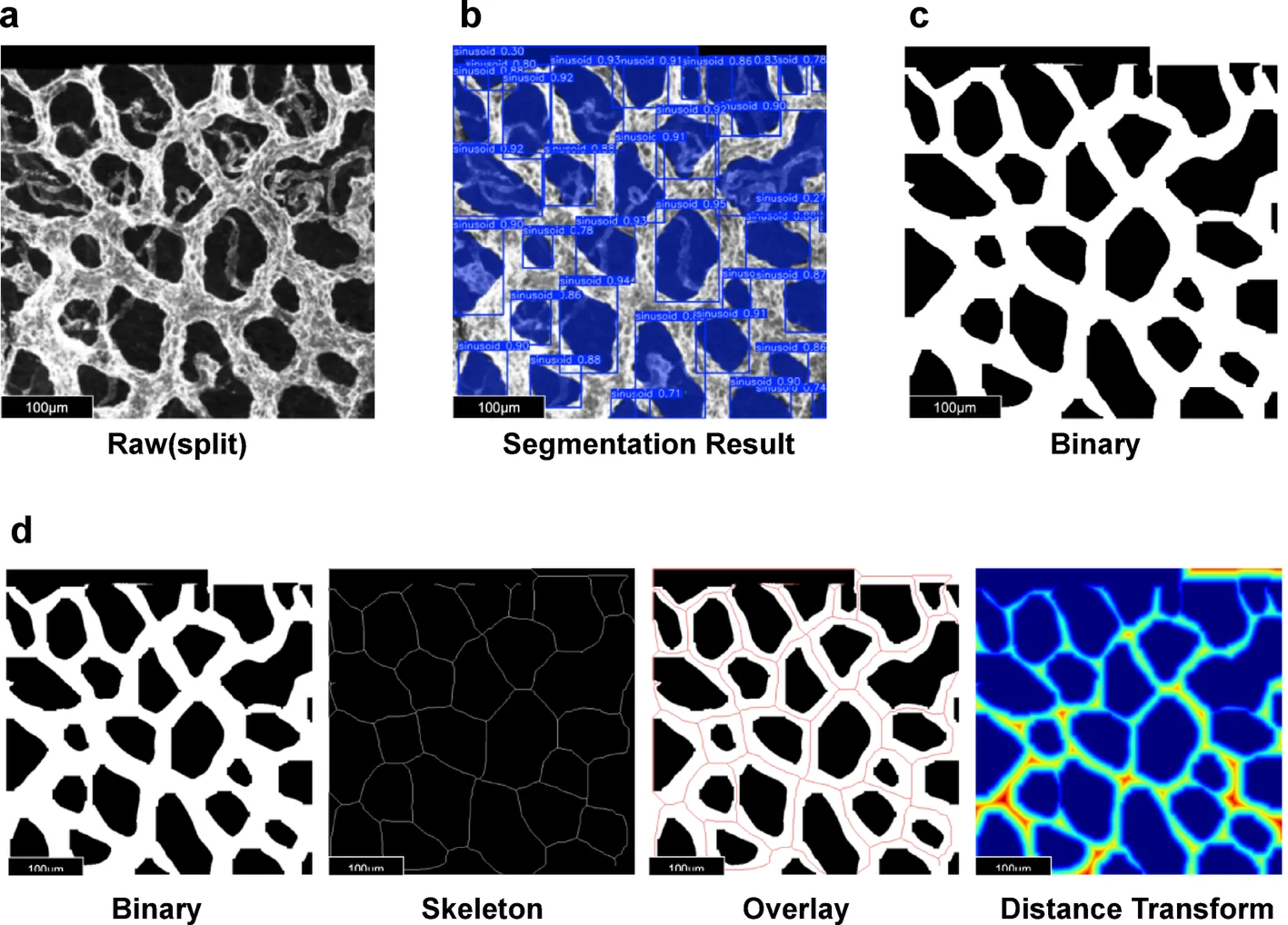
Image processing workflow. **a** Original vascular microscopy images. Images of the mouse nasal septum. Scale bar: 100 μm. **b** YOLOv8 segmentation output identifying non-vessel regions (sinusoids). **c** Binary image after adaptive thresholding. **d** Processing results (left to right): binary image, skeletonized vessel centerlines, overlay of the skeleton on the original image, and distance transform visualization

The image processing workflow progressed from original images to processed outputs (Fig. 3). Starting with the original confocal microscopy images of CD31-stained vascular structures (Fig. 3a), the trained model identified non-vessel regions, which were simultaneously used to indicate the vessel region (Fig. 3b), and then converted to binary format via adaptive thresholding (Fig. 3c). Subsequently, a skeletonization algorithm was used to reduce the vessels to their medial axes while preserving the network topology using the Zhang–Suen thinning algorithm (Fig. 3d, second panel). The complete processing sequence included a binary image, skeleton, overlay, and distance transform visualization (Fig. 3d).

### Branch point detection and vessel identification algorithm

**Fig. 4:**
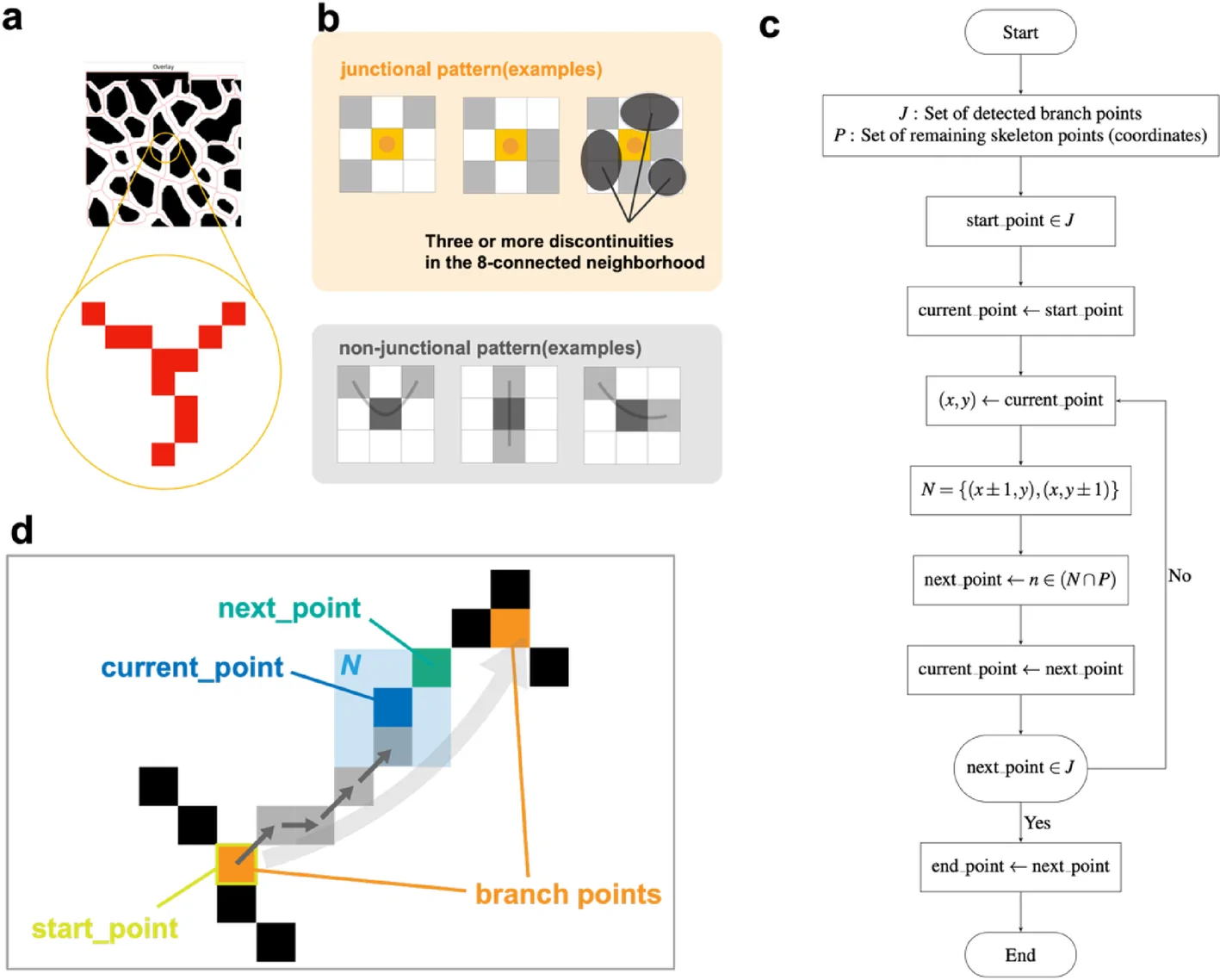
Branch point detection and vessel segment identification. **a** Detected branch points (highlighted in yellow) overlaid on the vessel skeleton, with an enlarged view of a representative junction pattern. **b** Classification of pixel patterns into non-junctional patterns (blue background, top) and junctional patterns (yellow background, bottom) for accurate branch point identification. **c** Algorithmic workflow for tracing vessel segments between branch points, showing the process of identifying distinct vessel paths. **d** Graphical illustration of the vessel-segment tracing concept, visually depicting how skeleton paths are followed between branch points to define distinct vessel segments

Algorithms were established to identify branch points and vessel segments within the vascular network. Local connectivity patterns in the skeleton were analyzed to detect junctions where vessels divided or merged by examining the 8-connected neighborhood of each skeleton pixel. Pixels with more than two connected neighbors that were not connected to each other were classified as branch-point candidates (Fig. 4a). Following branch point identification, a tracing algorithm identified distinct vessel segments connecting these junctions. The workflow for extracting the vessel segments is illustrated in a flowchart (Fig. 4c). This algorithm traces the vascular skeleton from each branch point to the next branch or terminal point, thereby decomposing the vascular network into discrete vessel segments with well-defined endpoints and path geometry (Fig. 4d). Pixels forming each segment were sequentially retrieved along the skeleton, allowing the central path of the vessel to be accurately reconstructed. This sequential pixel ordering is crucial for downstream diameter analysis. By preserving the connectivity and curvature of each vessel segment, the algorithm enabled reliable point-by-point measurements along the actual vessel path. Importantly, this is critical for accurately capturing the morphology of curved or tortuous vessels, where straight-line approximations introduce substantial error.

### Vessel diameter measurement

**Fig. 5:**
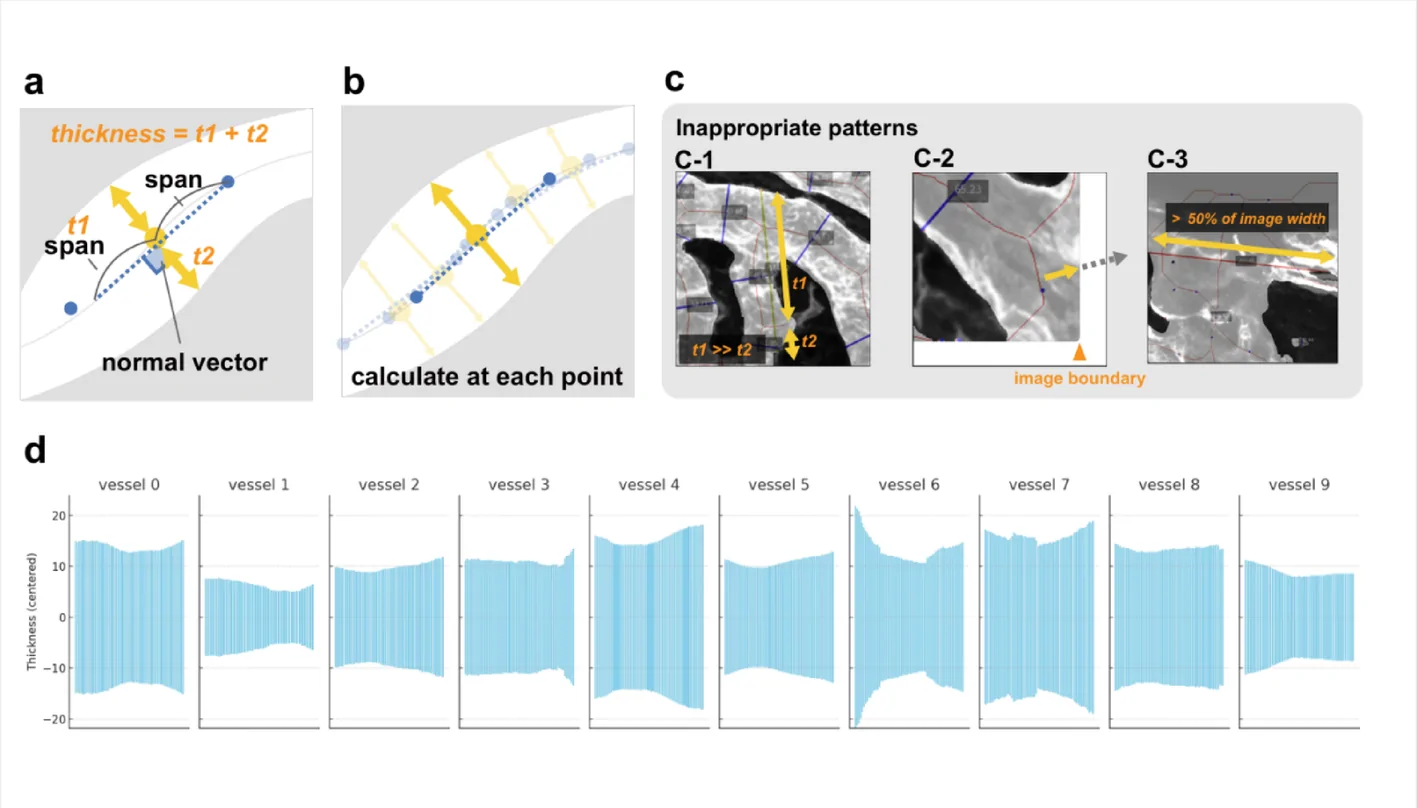
Vessel diameter measurement methodology. **a** Conceptual diagram illustrating diameter measurement perpendicular to the vessel direction, with the normal vector and measurement span indicated. **b** Measurement implementation at multiple points along the vessel segments to capture diameter variations. **c** Inappropriate measurement patterns that were automatically filtered: c-1 shows intersection with adjacent vessels, c-2 shows poorly defined boundaries, and c-3 shows complex branching patterns that confound the simple diameter measurements. **d** Representative diameter profiles along vessel segments, illustrating local variations in vessel caliber, including tapering and dilation, captured by the point-by-point perpendicular measurement approach

To overcome the limitations of conventional methods, which often assume straight vessels with uniform diameters, we developed an approach for measuring vessel diameters along anatomically accurate curved pathways. This methodology is illustrated in a conceptual diagram in Fig. 5. For each point along the vascular skeleton, the local vessel orientation was estimated by computing the tangent vector using a central difference approximation over a 10-pixel neighborhood (referred to as the “span”; Fig. 5a). Accordingly, a perpendicular line was drawn from the centerline, and the distance transform values were sampled in both directions. The distances to the vessel boundaries were determined using linear interpolation, and their sum was recorded as the vessel diameter at that point. Direction-specific measurements were performed for subsequent validation and quality control. Crucially, this computation was applied sequentially along the entire length of each vessel segment at 10-pixel intervals, enabling detailed point-by-point characterization of diameter variation (Fig. 5b). Measurements were taken at all points along the 25–75% range of the vessel pathway, with 50 measurements per vessel segment. Diameter measurements were strictly restricted to the interior portion of each vessel segment, excluding the immediate vicinity of branch points. Branch regions exhibit complex, non-tubular geometries in which multiple vessels intersect or diverge, thereby violating the assumption of a well-defined cross-sectional profile required for accurate diameter estimation. Accordingly, branch points were deemed unsuitable for diameter calculation and were intentionally excluded from quantitative diameter analysis. Importantly, branch points were preserved exclusively for defining vessel connectivity and constructing the graph-based network representation described below and were not used for diameter quantification. This functional separation enables reliable segment-wise diameter profiling while preserving accurate topological information for subsequent graph-based analysis. By preserving pixel-wise connectivity and following the vessel’s natural curvature, the algorithm enables accurate measurements that reflect the true morphology of each vessel segment. The resulting diameter profiles, displayed in Fig. 5d, capture local tapering, dilation, and other morphological features that would be lost with simplified linear assumptions. Filtering was performed under the following conditions:

1. A pattern of measurements in both directions with respect to the centerline, but with extreme discrepancies in the calculation results, i.e., a difference of two or more times, was considered unsuitable (occurred in 5.7% of the measurements; Fig. 5c-1)
2. The measured range is outside the screen (Fig. 5c-2)
3. A pattern in which the measured length is extremely long or short. In this study, those exceeding 50% of the screen were excluded as extremely long, and those less than 1% of the screen were excluded as extremely short (1.7% of measurements; Fig. 5c-3)
4. Those with an excessively large change in length along the blood vessels; variance exceeding 200 (10% of the measurements).
5. Inappropriate length-to-diameter ratio; those with length/thickness < 1 (5% of the measurements).

These filtering operations excluded approximately 23% of the initial measurements and greatly improved the reliability of the remaining data.

### Overlapping tiling and result integration

**Fig. 6:**
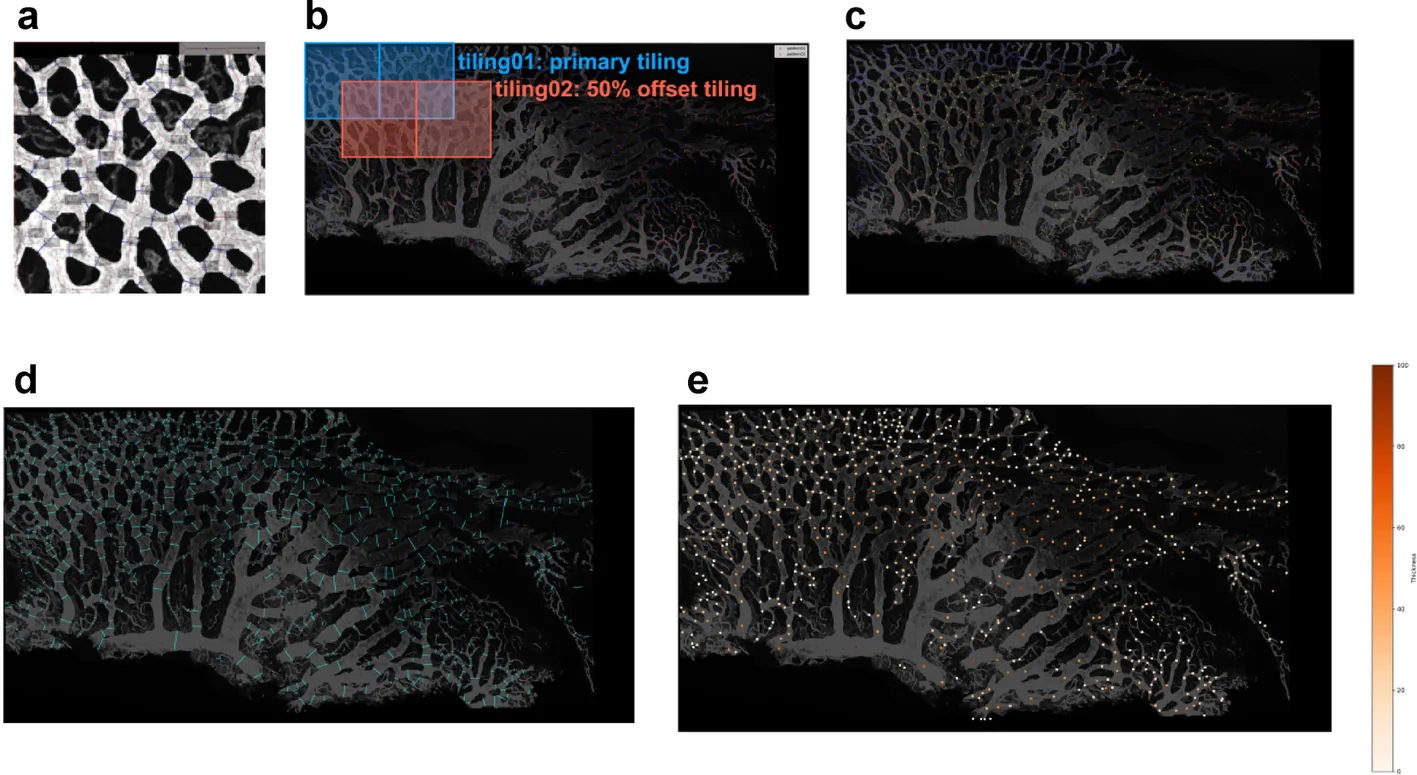
Overlapping tiling analysis approach for robust vessel detection. **a** Sample vascular image used for analysis. **b** Implementation of two tiling patterns (tiling01 in blue, tiling02 in red) for analyzing the same image. **c**, **d** Results of vessel tracing for each partitioning pattern independently, with identified vessel segments overlaid on the original image. **e** Integrated result with a heat map visualization of vessel diameters, where color intensity corresponds to vessel thickness

For large-field vascular images, tiling is necessary to enable computationally efficient processing. However, detection reliability decreases near tile edges due to truncated contextual information, which is particularly problematic for sinusoid structures that depend on local spatial continuity. To mitigate this systematic limitation, we implemented an overlapping tiling scheme with spatial offsets. The same image was processed multiple times using identical tile sizes but different spatial offsets, ensuring that each vascular structure appeared in the central region of at least one tile. As sinusoid detection is most reliable in the central tile region, this strategy ensured that every vessel was analyzed under optimal conditions at least once. The results obtained from the offset tiles were subsequently integrated to reconstruct continuous sinusoid structures across the entire image. Using this process, it was possible to identify all vessels within a large-sample image and calculate their diameters (Fig. 6d). Additionally, the integrated results are presented as a vessel diameter heatmap, in which the intensity of the color corresponds to the thickness of the vessels, offering insights into the overall trends (Fig. 6e). The integration algorithm matched corresponding vessels detected under different spatial offsets using a 50-pixel matching threshold, incorporating spatial proximity, orientation, and similarity in the calculated vessel diameters. This integration strategy substantially improved vessel detection by compensating for boundary-related omissions observed in single-tiling analyses.

### Graph-based representation and analysis

**Fig. 7:**
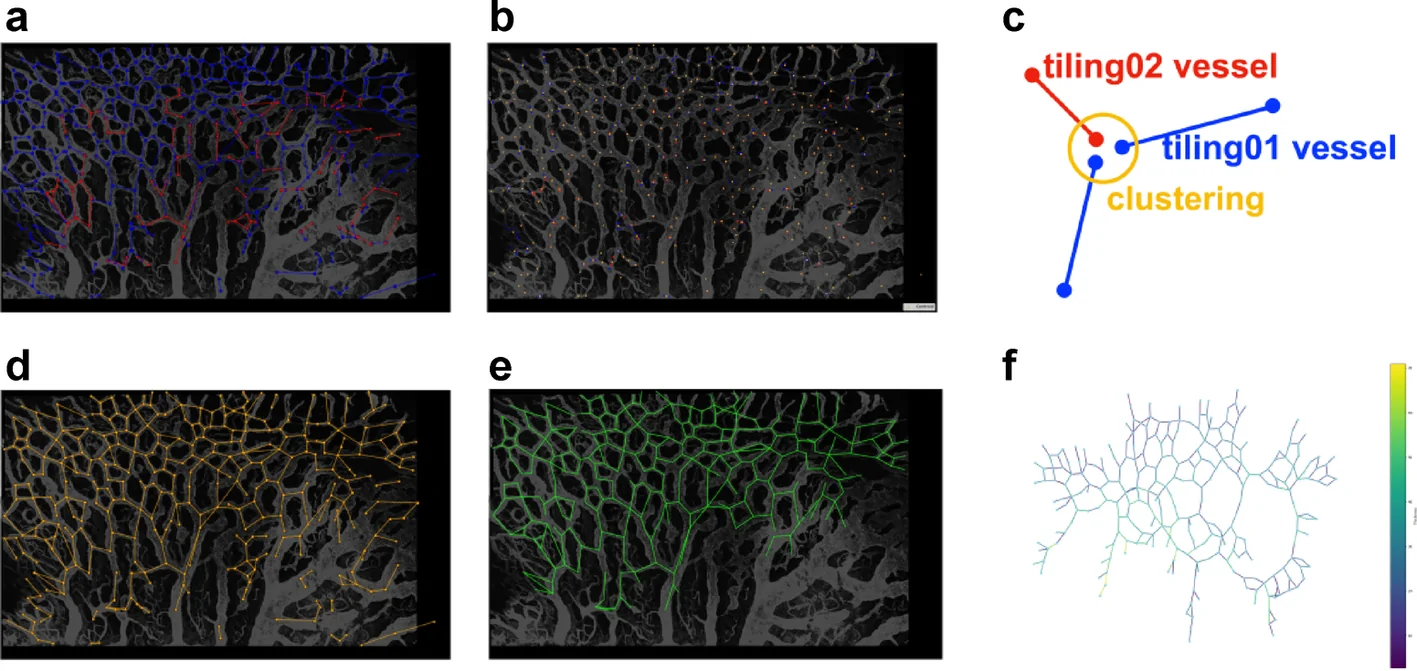
Graph-based representation of vascular network. **a** Vessel segments identified from both analysis patterns (tiling01 in blue, tiling02 in red). **b** Branch points (nodes) identified in the network. **c** Conceptual diagram of the graphical representation where tiling01 vessels (blue nodes), tiling02 vessels (red nodes), and their clustering (yellow highlight) are represented as nodes and edges. **d**, **e** Different visualizations of the complete vascular graph, highlighting the structural properties of the network. **f** Abstracted graph with color coding based on branch level or vessel diameter, illustrating the hierarchical organization of the vascular network

Methods for converting vascular structures into graph representations have been developed for quantitative network analysis. Vessel segments from both tilings (tiling01 in blue, tiling02 in red) served as the foundation for this representation (Fig. 7a). When identifying vessels, information on branch points and connectivity was preserved. Based on this information, the two terminal branch points of each vessel segment were determined and visualized as segments from both analysis tilings—tiling01 in blue and tiling02 in red (Fig. 7b). Because of the presence of small vessels missed during filtering, and the fact that in overlapping tiling analysis, vessels may appear connected in the image but lack common branch points, local clustering was employed to unify the branch points (Fig. 7c). Figure 7b displays the branch points before integration (blue for tiling01, red for tiling02), and the unified branch points after clustering in yellow. In this graphical representation, each branch point becomes a node, and each vessel segment represents an edge connecting two nodes. Vessel attributes, such as diameter, length, and tortuosity, were assigned as edge properties. The node degree distribution followed a power law relationship typical of biological networks, with most nodes having three connections (bifurcations) and fewer nodes having a higher connectivity (complex junctions). Different visualizations of the complete vascular graph display the network's structural properties (Fig. 7d and e). In Fig. 7e, the largest connected component of the graph was extracted, allowing peripheral vessel exclusion and focusing on a more relevant capillary network. Figure 7f presents this largest component using the Kamada-Kawai layout. This graph-based representation enables the quantitative analysis of network properties, including hierarchical metrics such as the average path length, clustering coefficient, and Strahler order.

### Tools and libraries

All image analyses were performed using Python 3.11 with the following key libraries: TensorFlow 2.5 for deep learning implementation [38], YOLOv8 for training segmentation data [39–41], OpenCV 4.13.0 for image processing [42], scikit-image 0.18 for morphological operations [43], and NetworkX 2.6 for graph-based analysis [44]. Numerical and statistical computations used NumPy (v1.26.4), pandas (v2.3.3), and SciPy (v1.11.4). Image input and processing were conducted with OpenCV (v4.13.0), scikit-image (v0.22.0), Pillow (v12.1.0), and tifffile (v2022.10.10). Visualization used Matplotlib (v3.8.0). Graph-based analysis employed NetworkX (v3.1), and clustering used scikit-learn (v1.2.2). Deep learning-based instance segmentation was performed with YOLOv8 implemented in the Ultralytics framework (v8.3.248).

## Results

### Run samples

The pipeline was applied to two confocal microscopy images that were not included in the training dataset. All analyses were performed on a MacBook Pro (14-inch, 2021) with 32 GB memory. For sample 1 (image size: 2223 × 1337 pixels), 406 vessel segments were identified (353 qualified). The average processing time per tile was 151.6 ms (preprocessing: 3.7 ms, inference: 127.7 ms, postprocessing: 20.1 ms; minimum–maximum: 103.7–218.7 ms). The total processing time up to vessel detection was 3.7 s, followed by 9.56 s for graph construction, with visualization requiring an additional 7.64 s. For sample 2 (image size: 1606 × 1362 pixels), 283 vessel segments were identified (238 qualified). The average processing time per tile was 109.4 ms (preprocessing: 3.1 ms, inference: 95.4 ms, postprocessing: 11.0 ms; minimum–maximum: 86.3–161.3 ms). The total processing time up to vessel detection was 2.6 s, followed by 7.81 s for graph construction, with visualization requiring an additional 4.8 s. These outputs were used for subsequent accuracy assessment against manual measurements.

### Validation of automated vessel detection and diameter measurement

**Fig. 8:**
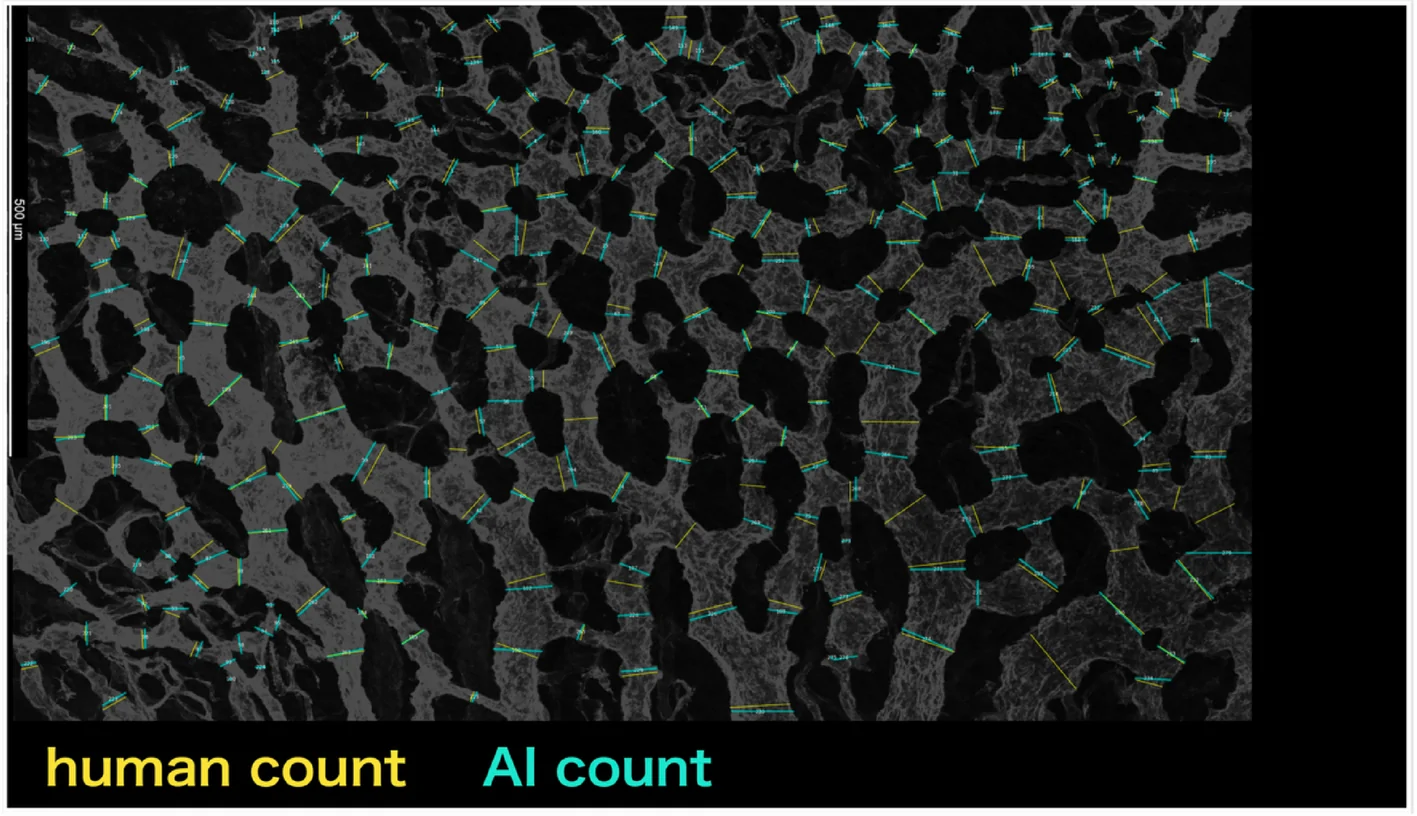
Validation of automated measurements against manual measurements. Comparison between human expert measurements (yellow) and AI-generated measurements (cyan) on the same vascular network, demonstrating concordance between manual and automated assessments for branch-point identification and vessel diameter measurement. Detection rates: 90.7% and 97.6% for two representative samples. AI, artificial intelligence

The automated analysis system was validated by comparison with expert manual annotations. Visual inspection demonstrated close concordance between human (yellow) and artificial intelligence (AI)-generated (cyan) measurements within the same vascular networks (Fig. 8). Most manually identified vessels and branch points were also detected by the automated system. At the detection level, the automated method achieved detection rates of 90.7% and 97.6% in two representative samples. Undetected vessels were primarily small peripheral segments or structures near complex branching regions. To evaluate quantitative accuracy, diameter comparisons were performed at the vessel-segment level. Paired statistical analyses were restricted to segments with both manual and automated diameter values; undetected vessels and false-positive detections were excluded to ensure one-to-one correspondence.

**Fig. 9:**
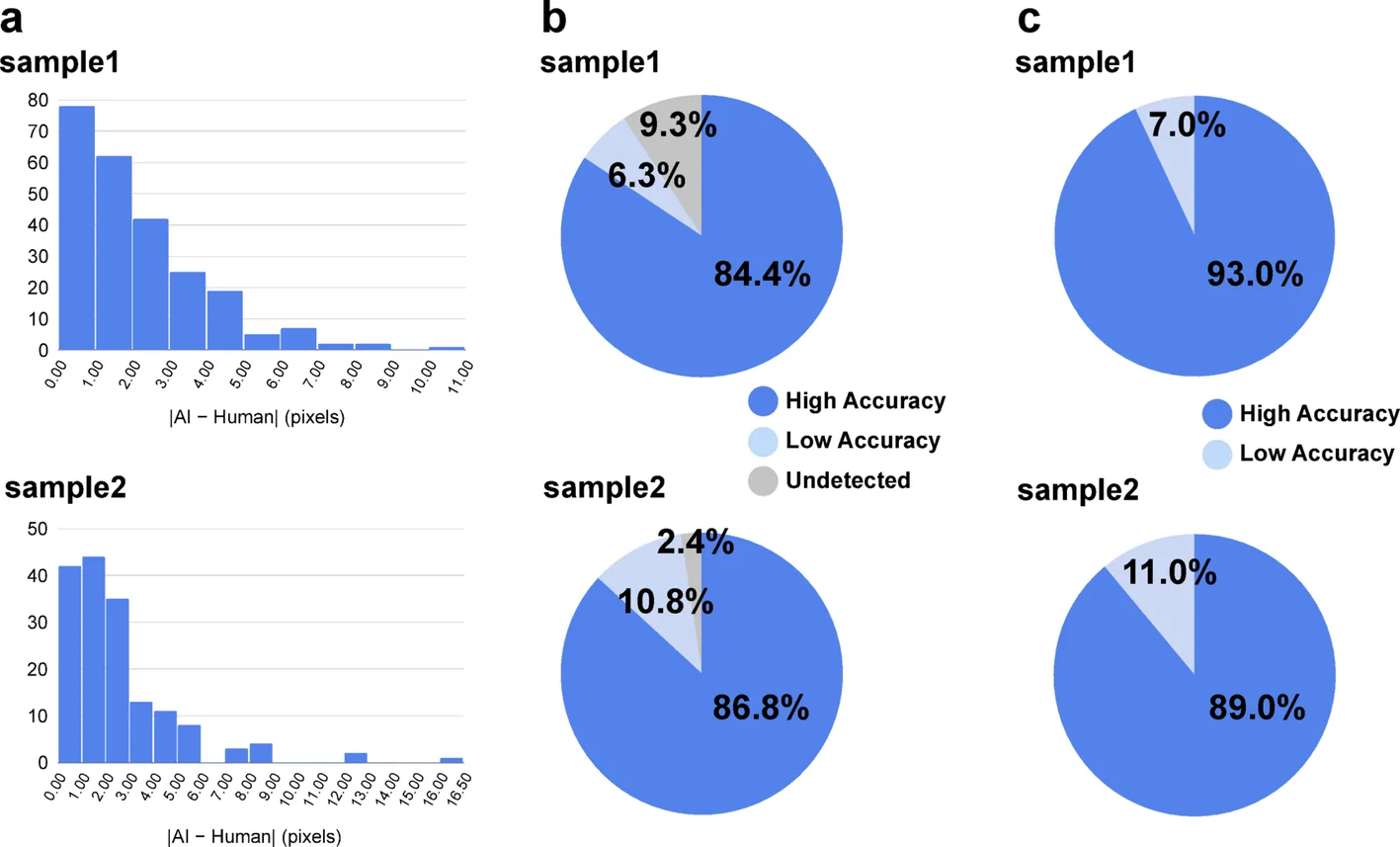
Statistical analysis of measurement accuracy. **a** Distribution of absolute differences between automated and manual measurements for two sample images, showing the frequency of deviation in different ranges. **b** Pie charts quantifying the accuracy levels for both samples, categorized into high accuracy (< 10 pixels, blue), low accuracy (10–20 pixels difference, light blue), and undetected vessels (gray). **c** Accuracy distribution when considering only detected vessels (excluding undetected vessels), exhibiting improved performance with high accuracy rates of 93.0% for sample 1 and 89.0% for sample 2

The distribution of absolute diameter differences was right-skewed, with most measurements within small margins (Fig. 9a). In sample 1, 84.4% of manually identified vessels were classified as highly accurate (difference < 10 pixels), 6.3% showed larger discrepancies, and 9.3% were undetected. In sample 2, 86.8% were highly accurate, 10.8% showed moderate discrepancies, and 2.4% were undetected. Restricting analysis to detected vessels increased the proportion of high-accuracy measurements to 93.0% and 89.0% for samples 1 and 2, respectively (Fig. 9c).

### Segment-wise paired statistical analysis

**Fig. 10:**
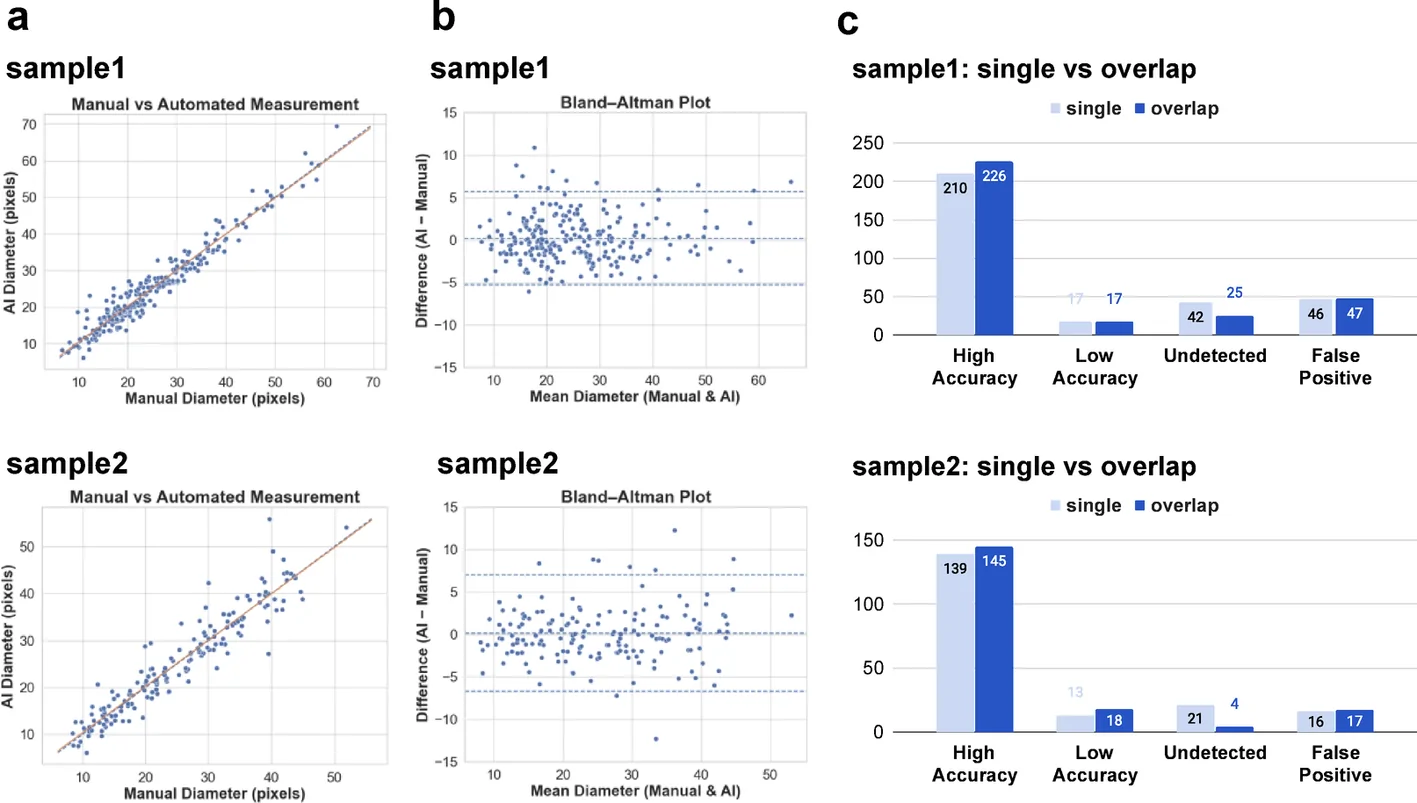
Segment-wise paired diameter validation and quantitative impact of overlapping tiling integration. **a** Paired comparison of automated (AI) and manual vessel diameter measurements for two independent samples. For sample 1, the mean difference between automated and manual measurements was + 0.208 pixels (95% CI, − 0.145 to 0.562), not significantly different from zero (paired t-test *p* = 0.247; Wilcoxon p = 0.886). For sample 2, a comparable result was obtained (mean difference =  + 0.141 pixels; 95% CI, − 0.400 to 0.681; paired t-test *p* = 0.608; Wilcoxon *p* = 0.888). **b** Correlation and agreement analyses between automated and manual measurements. Strong linear and rank correlations were observed (sample 1: Pearson r = 0.967, Spearman ρ = 0.944; sample 2: Pearson r = 0.945, Spearman ρ = 0.953). The Bland–Altman analysis demonstrated limits of agreement of − 5.280 to + 5.697 pixels (sample 1) and − 6.709 to + 6.990 pixels (sample 2). MAE was 2.129 pixels (median 1.646) for sample 1 and 2.490 pixels (median 1.918) for sample 2. **c** Ablation study of the overlapping tiling integration strategy. Compared with single-tiling analysis, overlapping integration increased total detected vessels by 7.0% (sample 1) and 7.2% (sample 2). High-accuracy detections increased by 7.6 and 4.3%, respectively, while undetected vessels decreased by 40.5% and 81.0%, respectively. Although false-positive detections increased (2.2% in sample 1; 6.3% in sample 2), the gain in true detections exceeded the increase in false positives, indicating a favorable trade-off between sensitivity and specificity. AI, artificial intelligence; CI, confidence interval; MAE, mean absolute error

For sample 1, the mean difference between automated and manual measurements was + 0.208 pixels (95% confidence interval [CI], − 0.145 to 0.562 pixels), which was not significantly different from zero (paired t-test *p* = 0.247; Wilcoxon *p* = 0.886) (Fig. 10a). The mean absolute error (MAE) was 2.129 pixels (median 1.646 pixels). The Bland–Altman analysis showed limits of agreement from − 5.280 to + 5.697 pixels. Strong linear and rank correlations were observed (Pearson r = 0.967, *p* = 1.19 × 10⁻^145^; Spearman ρ = 0.944, *p* = 4.00 × 10^−118^) (Fig. 10b). In sample 2, a similar pattern was observed. The mean difference was + 0.141 pixels (95% CI, − 0.400 to 0.681 pixels), likewise not significantly different from zero (paired t-test *p* = 0.608; Wilcoxon *p* = 0.888), with an MAE of 2.490 pixels (median 1.918 pixels) (Fig. 10a). The Bland–Altman limits of agreement ranged from − 6.709 to + 6.990 pixels. Correlation remained strong (Pearson r = 0.945, *p* = 5.88 × 10^−80^; Spearman ρ = 0.953, *p* = 2.80 × 10^−85^) (Fig. 10b). Across both independent samples, the automated approach exhibited no systematic bias (mean difference < 0.3 pixels, p > 0.2 for both paired t-test and Wilcoxon tests); limited absolute error (MAE < 2.5 pixels); strong linear correlation (r > 0.94); and strong preservation of relative diameter ranking (ρ > 0.94).

Although exact numerical equivalence was not achieved, automated measurements reliably captured segment-wise diameter variation suitable for comparative morphometric and graph-based network analyses.

### Ablation study of the overlapping tiling integration strategy

To quantitatively assess the contribution of the overlapping tiling integration strategy, we conducted an ablation study comparing single-tiling analysis (tiling01 only) with the proposed overlapping integration approach (tiling01 + tiling02 with a 50% offset).

For sample 1, overlapping integration increased the total number of detected vessels by 7.0% compared with single-tiling analysis. The number of high-accuracy detections increased by 7.6%, while low-accuracy detections remained unchanged (0.0% change). Importantly, the number of undetected vessels decreased by 40.5%. False-positive detections increased slightly by 2.2% (Fig. 10c).

For sample 2, a similar pattern was observed. The total number of detected vessels increased by 7.2%, with high-accuracy and low-accuracy detections increasing by 4.3% and 38.5%, respectively. Undetected vessels decreased significantly by 81.0%. False positives increased by 6.3%; however, the absolute increase remained limited relative to the gain in true detections (Fig. 10c).

Across both samples, the primary effect of overlapping integration was a substantial reduction in undetected vessels, resulting in improved detection coverage. Although a moderate increase in false positives was observed, the magnitude of this increase was smaller than the gain in true detections, indicating a favorable trade-off between sensitivity and specificity.

### Comparison with existing tools

**Fig. 11:**
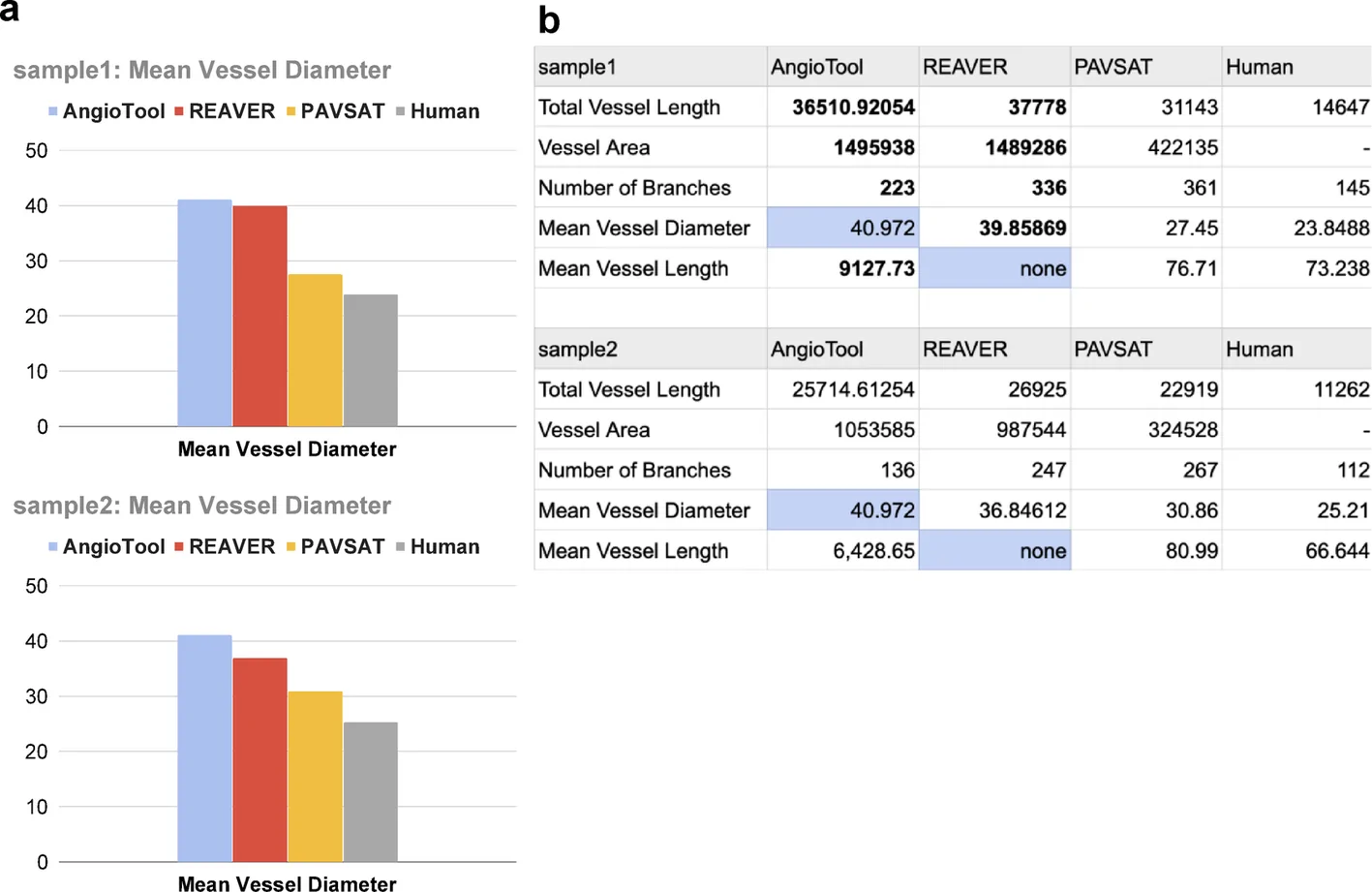
Quantitative comparison of PAVSAT with existing vascular analysis tools. **a** Bar graph showing mean vessel diameter measurements for two representative samples (sample 1 and sample 2) obtained using AngioTool, REAVER, PAVSAT, and expert manual measurements (Human). **b** Table summarizing quantitative outputs from each tool. AngioTool does not directly output mean vessel diameter; this value was calculated by dividing total vessel area by total vessel length. REAVER does not output mean vessel length. These parameters are highlighted with a blue background in the table. PAVSAT, Python-based Auto Vessel Segmentation Analysis Tool

To position PAVSAT within the landscape of existing vascular analysis software, we compared it with AngioTool [13] and REAVER [14], which are widely used for vessel segmentation and quantitative analysis (Figs. 11a, b). PAVSAT showed the smallest deviation from expert manual measurements in mean vessel diameter and mean vessel length. AngioTool does not directly output mean vessel diameter; thus, this value was calculated by dividing vessel area by total vessel length. REAVER does not output mean vessel length. Vessel area measurements from AngioTool and REAVER were comparable, suggesting similar segmentation coverage between these tools. Therefore, the primary distinction lies not in segmentation coverage alone but in how quantitative measurements are derived from segmented structures. AngioTool and REAVER compute global summary statistics from full-image segmentation masks and provide averaged values at the image or network level. Individual vessel segments are not explicitly decomposed or quantified. In contrast, PAVSAT decomposes the vascular network into discrete vessel segments between branch points and records individual segment properties, including length and diameter variation along curved centerlines. This segment-wise profiling reduces overestimation from global mask averaging and enables a more faithful representation of vessel morphology. Furthermore, while AngioTool and REAVER report branch points as numerical counts, PAVSAT preserves connectivity between branch points and vessel segments and exports them as graph network structures. This enables secondary graph-based network analyses, including degree distribution and hierarchical metrics, which are not directly supported in existing tools.

From an implementation perspective, AngioTool is distributed as a Windows executable, and REAVER requires MATLAB installation. In contrast, PAVSAT operates in a standard Python environment on consumer-grade hardware, providing operating system flexibility and broader accessibility. Outputs are generated in the standard CSV format, facilitating downstream workflows. Additionally, while AngioTool and REAVER rely on rule-based image processing for segmentation, PAVSAT employs a machine learning-based segmentation model. Pre-trained models are available, and users can fine-tune or deploy custom models for specific tissues or imaging conditions. These functional and architectural differences are summarized in Table 1.

**Table 1:** Feature comparison of vascular analysis software

	AngioTool	REAVER	PAVSAT
Detailed diameter calculation	× (average only)	× (average only)	◯
Graph network output	×	×	◯
Machine learning implementation	×	×	◯
OS-independent environment	× (Windows only)	△ (MATLAB)	◯

PAVSAT, Python-based Auto Vessel Segmentation Analysis Tool

### Additional validation on retinal vascular images

**Fig. 12:**
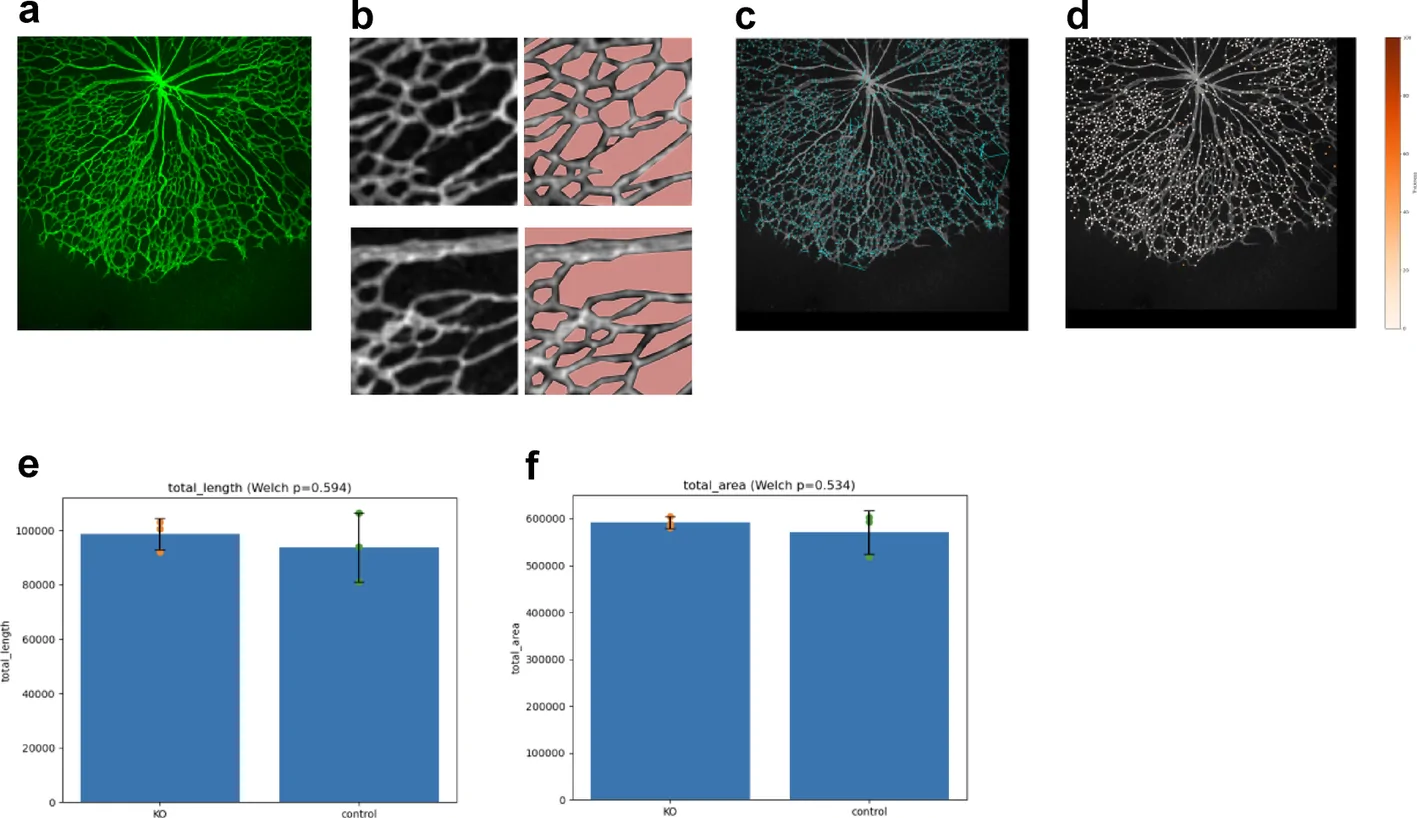
Application of PAVSAT to retinal vascular images. **a** Representative raw retinal vascular image obtained from a previously published WT and KO mouse study. **b** Manually annotated retinal image used for fine-tuning of the segmentation model. **c** Results of each vessel thickness on the target image. **d** Integrated result with a heat map visualization of vessel diameters, where color intensity corresponds to vessel thickness. **e** Bar graph showing the total vessel length quantified in WT and KO conditions. **f** Bar graph showing the total vessel area quantified in WT and KO conditions. PAVSAT, Python-based Auto Vessel Segmentation Analysis Tool; WT, wild type; KO, knockout

To further evaluate the adaptability of the framework, we applied the pipeline to retinal vascular images derived from a previously published study comparing wild-type (WT) and knockout (KO) mice [45] (Fig. 12a). Although the retinal vasculature is anatomically distinct from the nasal septum, it exhibits sinusoid-like intervascular spaces and a tubular vascular organization. From a representative retinal image, 30 non-overlapping tiles were generated and manually annotated for fine-tuning. Evaluation was subsequently performed on separate retinal regions that were not used during fine-tuning (Fig. 12b). Using the proposed pipeline, vessel area was quantified for both WT and KO conditions (Fig. 12c, d). The KO group showed a trend toward greater vascular area and total length than those of the WT group; however, these differences did not reach statistical significance in our dataset (vessel area: *p* = 0.534, total length: p = 0.594) (Fig. 12e, f). Notably, the direction of the observed effect was consistent with that in the original report, suggesting that the framework can reproduce previously reported trend-level vascular phenotypes when applied to structurally similar vascular networks. These results support the adaptability of the sinusoid-guided segmentation strategy while highlighting the need for larger sample sizes to draw definitive biological conclusions.

## Discussion

### Methodological contribution and conceptual positioning

The automated blood vessel analysis system developed in this study addresses fundamental challenges in quantitative vascular research by accurately measuring complex curved vessels with variable diameters and efficiently processing large image datasets. Although deep learning-based segmentation is employed as the front-end component, PAVSAT is not intended as a novel segmentation model. Instead, segmentation serves as an intermediate representation that enables precise geometric and topological quantification of vascular structures. The methodological contribution of this study lies in three key aspects. First, deep learning segmentation is used to robustly extract vascular masks under variable imaging conditions, providing a consistent structural basis for downstream analysis. Second, a curvature-aware perpendicular diameter estimation strategy was implemented. By computing local tangent vectors along the ordered skeleton and sampling perpendicular directions using distance transforms with filtering rules, the framework accurately captures local tapering and dilation. Diameter measurements are restricted to the central 25–75% of each vessel segment to avoid bifurcation artifacts, and unreliable measurements are automatically excluded. Third, vessel segments are explicitly defined between detected branch points, preserving anatomical connectivity. The vascular network is then transformed into a graph representation in which each segment becomes an attribute-rich edge. This enables higher-order network analyses, including hierarchical organization, clustering coefficients, Strahler order, and path length, that extend beyond conventional morphometric measurements. Collectively, these components establish PAVSAT as an integrated vascular geometry and topology analysis framework rather than a segmentation-focused methodology.

Validation against expert measurements demonstrated detection rates of 91–98% and diameter measurement accuracy of 89–93% within 10 pixels of manual measurements, with no detectable systematic bias between automated and manual diameters (mean difference < 0.3 pixels, paired t-test and Wilcoxon *p* > 0.2 for both samples) and strong linear and rank correlations (Pearson r > 0.94; Spearman ρ > 0.94). These results indicate performance approaching that of human experts but with significantly higher throughput, enabling large-scale vascular studies that would be impractical with conventional methods. The system identifies complex branching patterns, measures diameters along curved vessels, and generates comprehensive quantitative data suitable for statistical analysis. A key strength of this approach is its treatment of vascular geometry. Rather than treating vessels as linear structures with uniform diameters, the system accounts for their natural curvature and caliber variations. Moreover, graph-based representation transforms the image-based vascular data into a mathematical structure that facilitates higher-order network analysis, which is an increasingly important aspect of vascular function assessment that remains challenging with conventional methods. Despite its advantages, the system has limitations that should be considered to guide future development. Specifically, the deep learning component requires training data, which may be challenging to generate for new tissue types or imaging modalities. Complex vascular arrangements with overlapping vessels are difficult to analyze accurately. Future work should focus on extending the system to three-dimensional analysis and implementing transfer learning to reduce annotation requirements. This automated analytical approach can be applied to biomedical research. In developmental biology, it enables quantitative characterization of vascular network formation. In cancer research, tumor vasculature analysis may reveal patterns associated with progression and treatment response. In neuroscience, characterizing the brain's vascular network provides a framework for understanding neurovascular coupling and cerebrovascular pathologies. As imaging technologies continue to advance, computational tools, such as the one presented here, will become increasingly essential for extracting meaningful biological insights from complex vascular datasets.

### Segmentation architecture and model generality

Although YOLOv8 was employed for vessel segmentation in this study, the proposed pipeline is not inherently dependent on a specific segmentation architecture. Alternative models such as U-Net, nnU-Net, TransUNet, or SAM-based approaches may be appropriate depending on the imaging modality, tissue characteristics, and computational constraints. Beyond these established convolutional and transformer backbones, the medical image analysis field has recently undergone a rapid shift toward foundation models and vision-language models, which offer promising companion or successor solutions to conventional task-specific segmentation networks. Generalist foundation models trained on large-scale, heterogeneous medical image corpora have demonstrated the ability to perform open-world segmentation across diverse organs, modalities, and lesion types without task-specific fine-tuning [46], and extensions of the Segment Anything paradigm to three-dimensional medical volumes and videos have shown robust zero-shot and prompt-driven segmentation on varied anatomical targets [47], potentially reducing the annotation burden that currently limits deployment of task-specific models such as YOLOv8 to new tissues and imaging modalities. Complementary architectural advances in dense prediction, including scale-aware pyramidal feature learning [48], and label-efficient strategies based on weakly supervised spatiotemporal aggregation [49] highlight further avenues for reducing reliance on pixel-level expert annotations, which remain a key bottleneck for vascular microscopy. Generative approaches further broaden the methodological landscape: phase-to-fluorescence image translation using generative AI enables quantitative morphological analysis without dedicated fluorescent staining [50], while diffusion- and large-language-model-guided pipelines are being applied to general-purpose visual reconstruction [51], suggesting that multimodal generative frameworks could complement segmentation-based tools such as PAVSAT when raw imaging data are incomplete or weakly labeled. We regard these foundation and vision-language approaches as complementary rather than competitive to the present framework: PAVSAT’s geometric and graph-theoretical quantification layer operates downstream of segmentation and is agnostic to the segmentation backbone, so integrating foundation-model-derived or prompt-based segmentation outputs is a natural next step. As the present study focused on the development of an integrated analysis pipeline, comprehensive benchmarking of these alternative segmentation backbones was considered beyond its scope; however, systematic comparative evaluation on vascular microscopy data, together with exploration of multimodal and language-guided vascular analysis, represents an important direction for future research.

### Clinical and translational relevance

Beyond its methodological contributions, quantitative vascular analysis holds direct relevance to disease biology. Pathological vascular remodeling in tumor angiogenesis, chronic inflammatory disorders, and cerebrovascular disease is characterized not only by changes in vessel density but also by diameter heterogeneity, abnormal branching complexity, and altered network organization. For example, tumor vasculature often exhibits irregular calibers and disordered branching patterns that may partially normalize during anti-angiogenic therapy [4]. Similarly, inflammatory and ischemic tissues demonstrate remodeling patterns characterized by structural heterogeneity rather than uniform expansion or rarefaction [3, 9]. In this context, the ability of PAVSAT to generate segment-wise diameter distributions and graph-based network descriptors, such as node degree distribution, clustering coefficient, and hierarchical order, may provide quantitative measures that extend beyond conventional density-based metrics. Graph-theoretical representations of vascular systems have been increasingly explored in experimental and computational studies [1, 24], supporting the relevance of network-level descriptors in vascular biology. Although the present study emphasizes methodological development and validation, the framework is currently being applied in murine disease models, including inflammatory nasal tissue models, to evaluate vascular remodeling patterns. These preliminary applications indicate that the system is technically positioned for structured disease-model analysis; however, comprehensive clinical validation remains beyond the scope of this report.

### Limitations and future directions

This study has some limitations. First, the current implementation operates on two-dimensional confocal images. Although optical sectioning reduces background projection artifacts, signals from vessels below the focal plane may still influence the apparent surface-level architecture. In the present framework, this effect is partially mitigated by focusing on sinusoid regions defined by structurally dominant vessels. Signals from deeper or out-of-focus vessels typically appear diffuse or discontinuous and are incorporated into sinusoid annotations, reducing contamination of the primary network analysis. Nevertheless, because the analysis relies on single-plane images, complete suppression of depth-related artifacts cannot be ensured. Extension to volumetric (three-dimensional) imaging datasets represents an important future direction. Second, segmentation performance depends on task-specific training data. The current model was trained primarily on sinusoid-like vascular structures with mesh-like topology. Consequently, performance may degrade when applied to vessels with predominantly linear or strictly hierarchical organization. This limitation reflects structural bias in the training dataset rather than a limitation of the downstream quantification pipeline. Transfer learning or retraining will likely be required to adapt to new tissues or imaging modalities.

Third, diameter estimation near complex bifurcations remains challenging. To minimize branching artifacts, measurements were restricted to the central portion (25–75%) of each vessel segment. Although this strategy improves reliability, it may exclude morphological information adjacent to junctions. Future refinements may incorporate branch-aware geometric modeling to enable more comprehensive bifurcation analysis. Representative failure cases are provided in Additional file 2.

### Perspective

Taken together, this framework provides a reproducible and scalable approach to quantitative vascular morphology analysis. Integration with longitudinal and therapeutic intervention studies may further clarify its role in translational vascular research.

## Conclusions

We developed PAVSAT, a deep learning-based vessel analysis system that can be used in a Python environment. The system integrates YOLOv8 segmentation, advanced image processing, and graph-theoretical analysis to provide automated quantification of complex vascular structures. The system successfully identifies complex branching patterns, measures diameters along curved vessels, and generates quantitative data suitable for large-scale studies. Validation demonstrated detection rates of 91–98% and measurement accuracy of 89–93% within 10 pixels of expert measurements, with no detectable systematic bias and strong agreement (Pearson r > 0.94) between automated and manual diameters. The open-source implementation facilitates research in vascular biology, development, and pathology by enabling efficient, reproducible analysis of vascular networks.

## Supplementary Information

Below is the link to the electronic supplementary material.

Supplementary Material 1.

Supplementary Material 2.

## Data Availability

All source code developed for this study is available within the article and its supplementary files.The vascular image dataset used for training and validation of the PAVSAT system was obtained through collaboration with the Department of Otorhinolaryngology-Head & Neck Surgery, University of Fukui, and is part of a study currently under review (Shimizu A et al., manuscript in revision). The dataset is available from the corresponding author upon reasonable request.

## Abbreviations

AI: Artificial intelligence
CI: Confidence interval
Grad-CAM: Gradient-weighted class activation mapping
KO: Knockout
MAE: Mean absolute error
mAP: Mean average precision
MRA: Magnetic resonance angiography
PAVSAT: Python-based auto vessel segmentation analysis tool
WT: Wild type
