# Supplementary material for: PAVSAT: an automated blood vessel analysis tool using deep learning-based segmentation and image processing
Source: BMC Bioinformatics. 2026 Jun 22;27:211. doi: 10.1186/s12859-026-06505-0 (PMC13543378; doi:10.1186/s12859-026-06505-0)
Supplement: Supplementary file 1 [file 12859_2026_6505_MOESM1_ESM.pdf]

Additional file 1: Layer-wise feature visualization of the trained YOLOv8 model

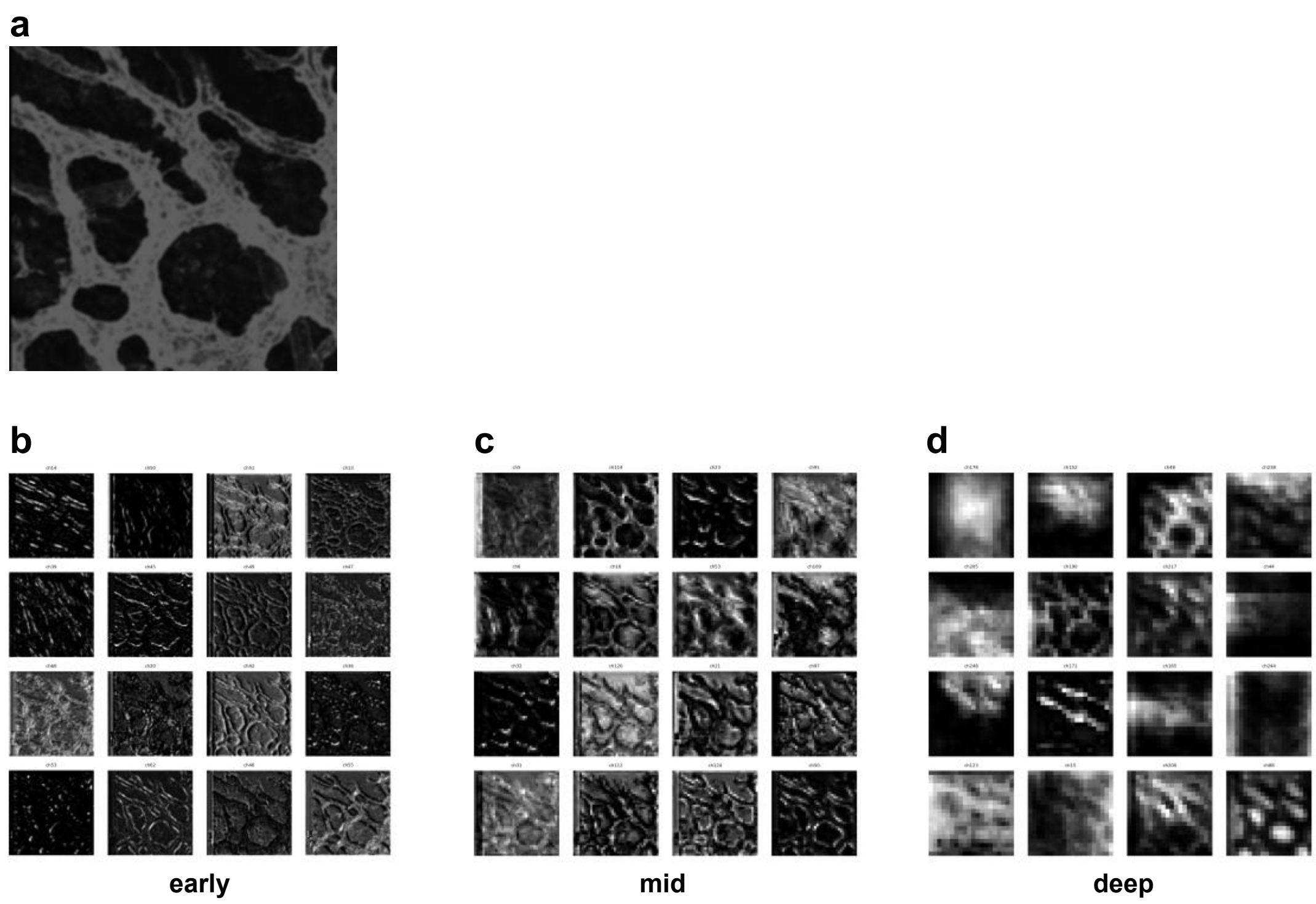

(a) Original confocal vascular image used for analysis. (b) Representative feature maps from an early convolutional layer, highlighting low-level edge and intensity gradients corresponding to vessel boundaries. (c) Representative feature maps from an intermediate layer, emphasizing elongated tubular structures. (d) Representative feature maps from a deeper layer, selectively responding to continuous vascular regions while suppressing background signals.

Additional file 2: Representative failure case from the REAVER Open Dataset (brain vasculature image)

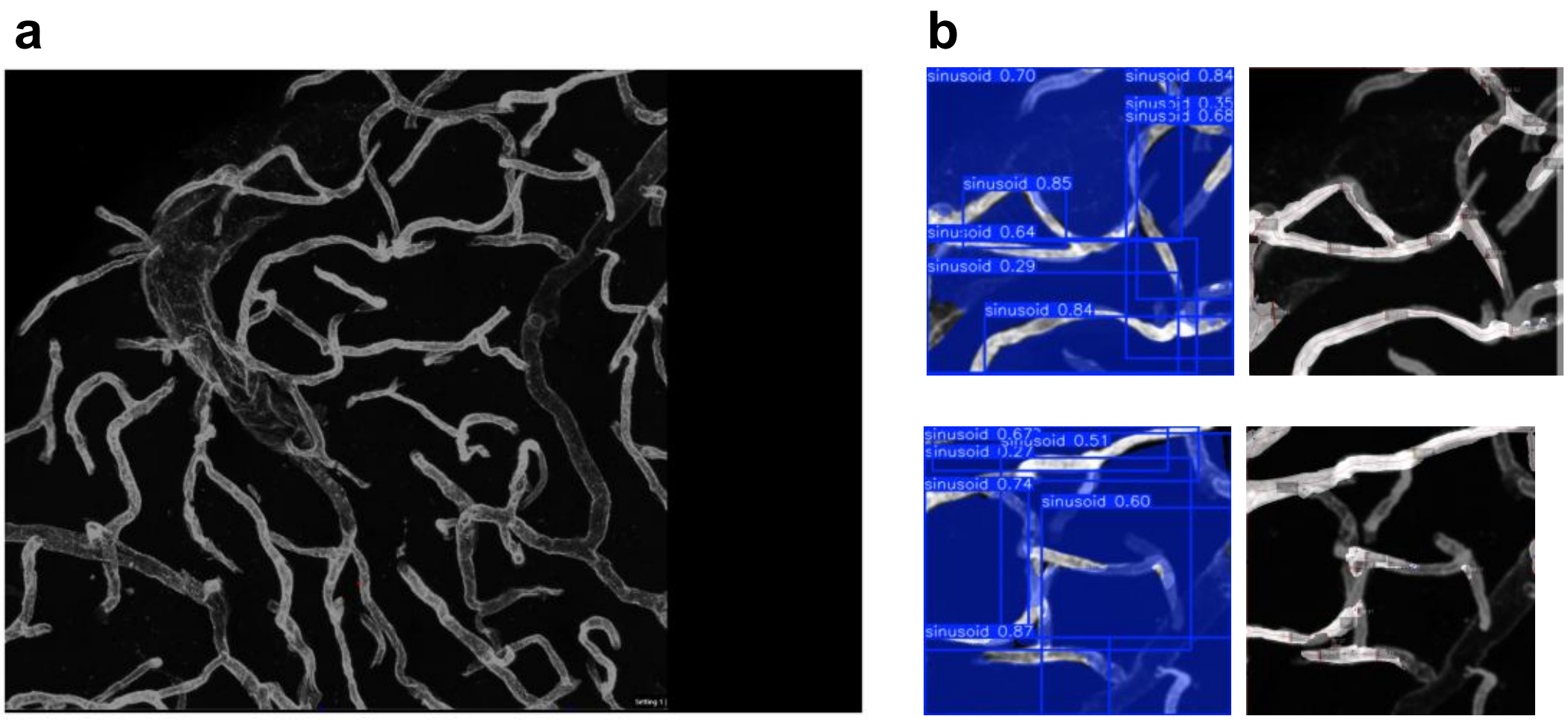

(a) Original fluorescence microscopy image from the REAVER Open Dataset. (b) Segmentation result from individual tiled images prior to integration, illustrating incomplete detection on the image lacking sinusoid structures.
